# Traumatische Milzverletzung mit unerwarteter Ruptur einer *Echinococcus*-Zyste: Sehr seltene Differenzialdiagnose im Schockraum

**DOI:** 10.1007/s00113-026-01707-w

**Published:** 2026-05-29

**Authors:** P. Schöttes, J. Richter, S. M. Schmidt-Dresely, P. Mertens, T. Strohmann, J.-P. Stahl

**Affiliations:** 1 Fakultät für Gesundheit, Universität Witten/Herdecke, Witten, Deutschland; 2 Klinik für Unfall‑, Hand- und Wiederherstellungschirurgie, Klinikum Dortmund gGmbH, Münsterstr. 240, 44145 Dortmund, Deutschland

**Keywords:** Hydatidzyste, Parasitäre Milzerkrankung, Schockraumdiagnostik, Milzlazeration, Anaphylaxierisiko, Hydatid cyst, Parasitic splenic disease, Emergency room diagnostics, Splenic laceration, Anaphylactic risk

## Abstract

Wir berichten über einen seltenen Fall einer traumatischen Milzruptur mit gleichzeitiger Ruptur einer intraparenchymatösen *Echinococcus*-Zyste bei einem 48-jährigen Patienten nach Leitersturz aus ca. 3 m Höhe. Die initiale Schockraumdiagnostik gemäß ATLS-Standard ergab sonographisch einen perisplenischen Flüssigkeitssaum sowie eine atypische, teilweise verkalkte Struktur im Milzparenchym. In der kontrastmittelgestützten Computertomographie (CT) zeigten sich eine hochgradige Milzlazeration mit massiver intraabdomineller Blutung sowie eine große kalzifizierte Zyste mit einem Volumen von 265 ml im kaudalen Milzpol mit Rupturzeichen. Trotz initial stabiler Kreislaufverhältnisse wurde aufgrund des Verletzungsausmaßes die Indikation zur sofortigen operativen Versorgung gestellt. Intraoperativ bestätigten sich die CT-Befunde; die Splenektomie mit Lavage war indiziert. Die histopathologische Untersuchung bestätigte eine rupturierte intraparenchymatöse *Echinococcus*-Zyste. Postoperativ erfolgten deshalb eine antiparasitäre Therapie mit Albendazol sowie die empfohlene Impfprophylaxe nach Splenektomie. Der Patient konnte nach 8 Tagen entlassen werden. Der Fall unterstreicht, dass auch in nichtendemischen Regionen bei Milzverletzungen mit atypischen zystischen oder kalzifizierten Befunden seltene parasitäre Erkrankungen differenzialdiagnostisch berücksichtigt werden sollten. Die therapeutische Entscheidung im Akuttrauma richtet sich dann nicht nur nach dem hämodynamischen Status, sondern berücksichtigt das gesamte Verletzungsausmaß.

## Abstract (en)

We report a rare case of traumatic splenic rupture with concomitant rupture of an intraparenchymal echinococcal cyst in a 48-year-old male patient following a fall from a ladder from a height of approximately 3 m. The initial advance trauma life support (ATLS)-based emergency room diagnostics revealed perisplenic free fluid on ultrasound examination as well as an atypical, partially calcified lesion within the splenic parenchyma. Contrast-enhanced computed tomography (CT) demonstrated a high-grade splenic laceration with massive intra-abdominal hemorrhage and a ruptured, calcified cyst located at the inferior splenic pole with a volume of approximately 265 ml. Despite initially stable hemodynamic parameters, immediate surgical intervention was indicated due to the extent of the injury. The CT findings were confirmed intraoperatively and splenectomy with abdominal lavage was performed. Histopathological examination verified a ruptured intraparenchymal echinococcal cyst. Postoperatively, antiparasitic treatment with albendazole was initiated and standard postsplenectomy vaccination was administered. The patient was discharged after 8 days. This case highlights that even in nonendemic regions, rare parasitic diseases should be considered in the differential diagnosis of splenic injuries with atypical cystic or calcified findings. In acute trauma settings, therapeutic decision-making should be guided not only by the hemodynamic status but also by the overall extent of injury.

## Falldarstellung

Ein 48-jähriger Handwerker stürzte während der Arbeit aus einer Höhe von etwa 3 m von einer Leiter und schlug auf die linke Körperhälfte auf. Die Rettung transportierte ihn bodengebunden.

**Abb. 1:**
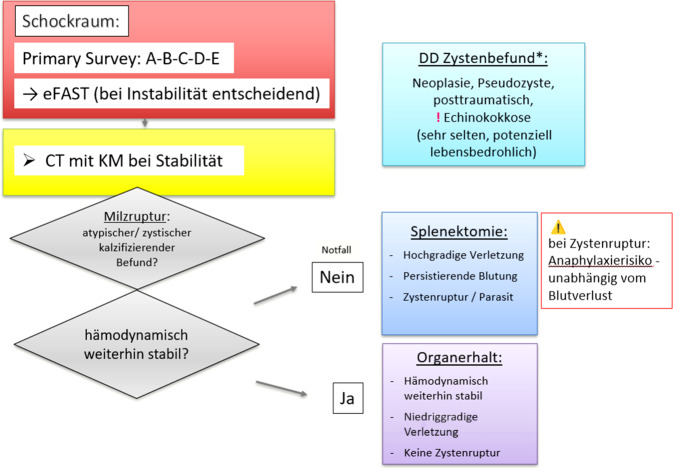
Der Advanced Trauma Life Support(ATLS)-basierte Algorithmus zur Versorgung von Patienten mit Abdominaltrauma und Milzverletzung zeigt: Bei stabilen Patienten erlaubt die CT-Diagnostik die Identifikation atypischer zystischer Milzbefunde. Die Therapieentscheidung richtet sich primär nach dem hämodynamischen Status. Eine Parasitenzystenruptur kann in diesem Zusammenhang eine Anaphylaxie auslösen. (*Asterisk* vgl. Abb. 2 und Tab. 1)

Beim Eintreffen im Schockraum (Abb. 1 und 2) war der Patient wach, reagierte auf Ansprache und war initial hämodynamisch stabil. Klinisch bestanden Druckschmerzen im linken Oberbauch sowie eine Prellmarke an der linken Flanke. Nebenbefundlich zeigten sich eine Schädelprellung sowie eine Thoraxprellung mit diskreter Lungenkontusion und eine unverschobene Radiusfraktur (AO-Typ 2R3-B1.1), die konservativ behandelt werden konnten.

**Abb. 2:**
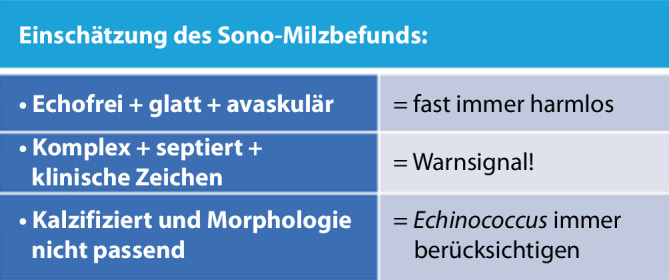
Merksätze für den Schockraum zur Milzbeurteilung

**Abb. 3:**
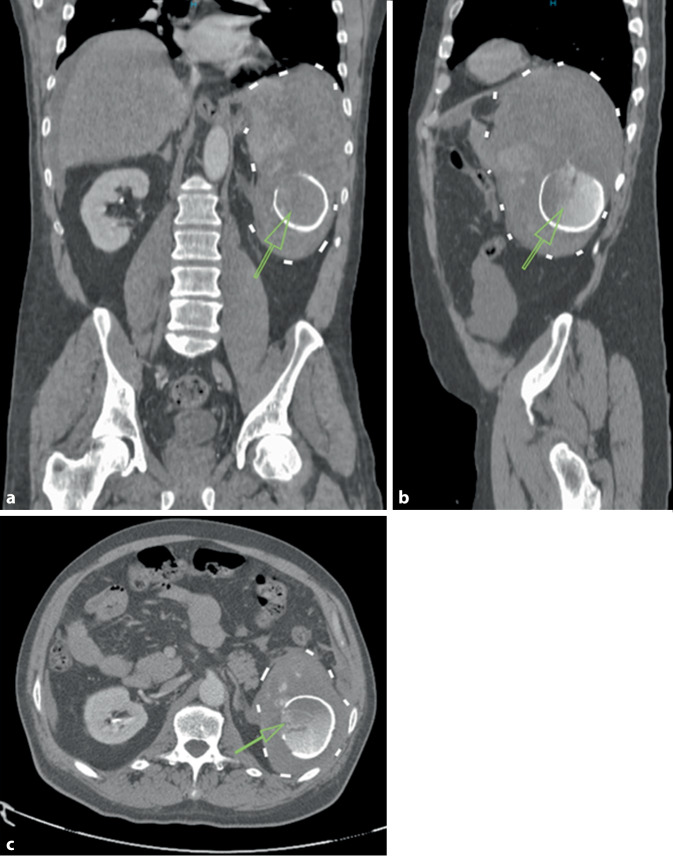
**a–c** Kontrastmittel-CT des Abdomens: (**a**) Koronare CT-Rekonstruktion mit hochgradiger Milzlazeration und perisplenischem Hämatom (*weiß*-*gestrichelt*). (**b**) Sagittale Darstellung mit rupturierter kalzifizierter Zyste (*grüner Pfeil*). (**c**) Axiale Schnittdarstellung der Zyste. Das Volumen beträgt 265 ml, Typ-IV-Verletzung der Milz nach American Association for the Surgery of Trauma (*AAST*) – splenic injury scale

Die eFAST-Sonographie ergab einen perisplenischen Flüssigkeitssaum sowie eine atypische, verkalkte Struktur im Milzparenchym. Die kontrastmittelgestützte Computertomographie nach Polytraumaprotokoll wies eine IV-gradige Milzlazeration [1] mit massiver intraabdomineller Blutung nach. Zusätzlich fand sich im kaudalen Milzpol eine bis zu 265 ml große, kalzifizierte Zyste mit Hinweis auf eine hiluswärts gerichtete Ruptur [2]. Weitere intraabdominelle Verletzungen bestanden nicht (Abb. 3).

Aufgrund des Verletzungsausmaßes in Kombination mit der Zystenruptur wurde trotz initial stabiler Kreislaufverhältnisse die Indikation zur sofortigen Splenektomie mit Abdomenlavage gestellt [3].

Intraoperativ zeigte sich eine vollständig fragmentierte Milz, die in mehrere Teilstücke zerrissen war. Der kaudale Milzpol enthielt eine etwa faustgroße, stark verkalkte *intraparenchymatöse* Zyste, *deren Kapsel vollständig* eröffnet war. *Es fanden sich Hinweise auf den Austritt von Zysteninhalt im Rahmen der traumatischen Organzerstörung*. Es bestand eine ausgeprägte intraabdominelle Blutung, sodass eine Transfusion von zwei Erythrozytenkonzentraten (EK) und zwei Einheiten Fresh Frozen Plasma (FFP) erforderlich wurde. Auch aufgrund der ausgedehnten Organzerstörung war ein organerhaltendes Vorgehen nicht möglich.

Postoperativ wurde der Patient extubiert und für 32 h intensivmedizinisch überwacht. In diesem Zusammenhang wurden zwei weitere EK transfundiert. Danach konnte er auf die Normalstation verlegt werden. Der weitere Verlauf war *komplikationslos*. Die histopathologische Untersuchung bestätigte eine rupturierte intraparenchymatöse *Echinococcus-*Zyste.

Die empfohlene Impfprophylaxe nach Splenektomie zur Prävention eines „*Overwhelming Post Splenectomy Infection* (OPSI) Syndrome“, einschließlich Pneumokokkenimpfung, erfolgte am vierten postoperativen Tag. Ergänzend wurde eine antiparasitäre Therapie mit Albendazol (2-mal 400 mg täglich) über vier Wochen eingeleitet. *Es erfolgte* eine infektiologische *Mitbetreuung* mit *serologischen* Verlaufskontrollen. *Zusätzlich wurde* eine bildgebende Kontrolle des Abdomens empfohlen, um eine sekundäre peritoneale Hydatidose auszuschließen. *Bis zum Zeitpunkt der letzten Nachuntersuchung ergaben sich hierfür keine Hinweise.* Der Patient konnte am siebten postoperativen Tag in gutem Allgemeinzustand entlassen werden.

## Diskussion

Die zystische Echinokokkose ist in Deutschland selten und betrifft die Milz nur in einem kleinen Anteil der Fälle [3]. *In der deutschsprachigen traumaspezifischen Literatur stellt eine traumatische Ruptur einer splenischen Echinococcus-Zyste eine Rarität dar*. *Entsprechend gering ist die klinische Erfahrung mit splenischen Echinococcus-Zysten im unfallchirurgischen Alltag*.

Der vorliegende Fall zeigt eine außergewöhnliche, für den Schockraum jedoch relevante Konstellation, bei der eine bislang asymptomatische intraparenchymatöse *Echinococcus*-Zyste im Rahmen eines stumpfen Abdominaltraumas mi einer hochgradigen Milzlazeration einherging. *Insbesondere verkalkte oder komplexe zystische Milzbefunde sollten in der Akutsituation nicht vorschnell als harmlose Zufallsbefunde interpretiert, sondern proaktiv relevanten Differentialdiagnosen zugeordnet werden*.

Die Bildgebung spielt hierbei eine zentrale Rolle [2, 4]. Während die Sonographie Hinweise auf eine Milzverletzung und einen atypischen Befund liefern kann, ermöglicht erst die kontrastmittelgestützte Computertomographie eine vollständige Beurteilung des Verletzungsausmaßes und *der Morphologie zystischer Läsionen*. *Die strukturierte Anwendung eines ATLS-basierten Schockraumalgorithmus unterstützt dabei die frühzeitige Identifikation solcher Sonderbefunde und deren korrekte klinische Einordnung *(Tab. 1; [5, 6]).

**Tab. 1:** Zufällig entdeckte zystische Läsionen der Milz in Sonographie oder CT für den Schockraum-Algorithmus.

Entität	Sonographie (B-Bild ± Doppler)	CT-Morphologie	Relative Häufigkeit
Kongenital (epithelial)	Rund/oval, glatt begrenzt, echofrei, dorsale Schallverstärkung, avaskulär	Homogen hypodens, dünne Wand, keine KM-Aufnahme	Häufig (ca. 40–60 % der nichtparasitären Zysten)
Posttraumatisch	Echoarm bis echoreich, oft Wandverkalkungen, avaskulär	Hypodens, häufig randständige Verkalkungen	Häufig (großer Anteil der sekundären Zysten)
*Echinococcus* Zyste	Komplex, Septen, Membranen, „Schneegestöber“	Mehrkammerig, evtl. Tochterzysten, Verkalkungen	Sehr selten (außer Endemiegebiete)
Milzabszess (bakteriell/mykotisch)	Echoarm bis komplex, ggf. Gasartefakte	Hypodens, dickwandig, KM-Randanreicherung	Selten (≈5–10 % der zystischen Läsionen)
Benigner vaskulärer Tumor (Hämangiom/Lymphangiom)	Inhomogen, teils zystische Anteile, variabel	Hypodens, evtl. KM-Enhancement solider Anteile	Selten
Zystische Metastase	Meist komplex, echoarm	Hypodens, irreguläre Wand, evtl. KM-Aufnahme	Extrem selten
Infarkt mit zystischer Einschmelzung	Keilförmig, echoarm	Hypodens, keilförmig	Selten

Fallberichte belegen, dass traumatische Rupturen hydatider Zysten auch nach moderaten Traumata auftreten können [7]. Von besonderer klinischer Bedeutung ist das Risiko einer akuten anaphylaktischen Reaktion infolge der Zystenruptur, die unabhängig von der initialen hämodynamischen Stabilität auftreten kann [8]. Dieser Aspekt ist insbesondere bei der operativen Entscheidungsfindung relevant [9].

*Bei hämodynamisch stabilen Patienten mit höhergradigen Milzverletzungen stellt die selektive Milzarterienembolisation grundsätzlich eine mögliche organerhaltende Therapieoption dar. Im vorliegenden Fall war dieses Vorgehen aufgrund der vollständigen Organfragmentierung, der massiven intraabdominellen Blutung sowie der rupturierten parasitären Zyste mit potenziellem Anaphylaxierisiko nicht indiziert.*

Im vorliegenden Fall zeigte sich intraoperativ eine vollständig fragmentierte Milz mit massiver Blutung, sodass ein organerhaltendes Vorgehen ausgeschlossen war. Die Splenektomie stellte sowohl zur Blutungskontrolle als auch zur Vermeidung einer weiteren parasitären Dissemination das sicherste Verfahren dar [10]. Um die systemische Ausbreitung der Parasiten zu verhindern, sollte auf die Nutzung eines Cell-Savers verzichtet werden [10, 11]. Die postoperative antiparasitäre Therapie sowie die *strukturierte* infektiologische Nachsorge sind essenziell, um das Risiko einer sekundären peritonealen Hydatidose zu minimieren.

Zusammenfassend verdeutlicht dieser Fall, dass seltene parasitäre Erkrankungen bei Milzverletzungen mit atypischen oder *kalzifizierten* Befunden *auch in nichtendemischen Regionen berücksichtigt werden sollten*. *Neben häufigeren Differenzialdiagnosen wie posttraumatischen oder kongenitalen Zysten müssen insbesondere bei komplexer Morphologie parasitäre Ursachen mitgedacht werden*. Die Therapieentscheidung im Akuttrauma muss sich konsequent am Verletzungsausmaß orientieren, wobei bei Sonderbefunden spezifische Anpassungen erforderlich sind.

### Limitationen

Als Einzelfalldarstellung ist die Generalisierbarkeit eingeschränkt. Trotzdem ist das hier vorgestellte Management auf andere interdisziplinäre Strukturen und Versorgungszentren übertragbar.

## Kernaussagen für die Praxis

- *Die Kombination aus Milzverletzung und atypischer oder kalzifizierter Zyste erfordert eine erweiterte differenzialdiagnostische Betrachtung*.
- Auch in nichtendemischen Regionen sollte bei komplexen zystischen Milzbefunden an eine Echinokokkose gedacht werden.
- Bei rupturierter parasitärer Zyste ist das Anaphylaxierisiko unabhängig von der Hämodynamik.
- *Eine angiographische Embolisation der Milz ist nur bei ausgewählten stabilen Patienten ohne intraparenchymatöse Zystenruptur eine Therapieoption*.
- Eine postoperative antiparasitäre Therapie und Postsplenektomie-Impfung sowie eine strukturierte Nachsorge sind obligater Bestandteil des Behandlungskonzepts.

## Data Availability

Die im Rahmen dieser Fallbeschreibung erhobenen Daten sind im Artikel enthalten.
